# Quantitative longitudinal photoacoustic imaging of vascular oxygenation for therapy response monitoring in Non-Hodgkin lymphoma

**DOI:** 10.1016/j.pacs.2026.100835

**Published:** 2026-05-13

**Authors:** Jing Shi, Ying Fan, Jie Mi, Qianping Mao, Yan Huang, Dean Ta, Ting Feng, Lin Chen

**Affiliations:** a Department of Ultrsound, Huadong Hospital Affiliated to Fudan University, Shanghai 200040, China; b College of Biomedical Engineering, Fudan University, Shanghai 200433, China

**Keywords:** Photoacoustic imaging, Non-Hodgkin lymphoma, Therapeutic response, Tumor vasculature remodeling, Hypoxia

## Abstract

Non-Hodgkin lymphoma (NHL) is clinically heterogeneous and lacks practical tools for repeated, noninvasive monitoring of response. We performed longitudinal photoacoustic imaging (PAI) at five time points in NHL xenografts to quantify Hb, HbO2, HbT, and mSO2, with endpoint histologic and serum validation. Untreated tumors exhibited a natural progression characterized by a progressive decline in mSO2. While Cyclophosphamide (CTX) slowed growth, it induced earlier alterations in oxygenation: mSO2 was higher in the CTX group on Day 7 (*P* < 0.05), before tumor volume divergence became evident, whereas Hb and HbT were lower at the mid-treatment stage. Thereafter, mSO2 declined more steeply in the CTX group from Day 7 to Day 13, despite similar absolute mSO2 values at the endpoint. Endpoint readouts showed higher HIF-1α and MVD, and lower VMI, indicating immature vasculature. These data support the use of longitudinal PAI as an additional biomarker to evaluate and optimize therapy in NHL.

## 1 Introduction

Non-Hodgkin lymphoma (NHL) comprises a heterogeneous group of lymphoid malignancies, accounting for nearly 90% of all lymphomas worldwide, with rising incidence and increasing global burden [1], [2]. Despite advances in targeted and immune therapies, including randomized evidence supporting improved outcomes across aggressive and indolent subtypes [3], [4] and durable responses to Bruton tyrosine kinase (BTK) inhibitors in mantle cell lymphoma, clinical outcomes remain highly variable [5], [6], [7]. This heterogeneity reflects complex interactions among tumor genomics, host immunity, pharmacogenomics, and the tumor microenvironment [8], [9], [10]. Among microenvironmental factors, hypoxia is increasingly recognized as a key determinant: it drives drug resistance and relapse in aggressive NHL, and may facilitate immune evasion and histologic transformation in indolent disease [11], [12]. While multiple studies have associated higher hypoxia with inferior progression-free and overall survival [9], [11], [13], others report unexpectedly favorable outcomes with hypoxia-inducible factor-1 alpha (HIF-1α) expression under R-CHOP therapy [14], underscoring the need for noninvasive, repeatable methods to functionally assess tumor oxygenation and clarify its prognostic relevance.

Current clinical tools for evaluating treatment response in NHL remain limited. Histological and serological markers are invasive and poorly tolerated in immunocompromised or elderly patients, particularly during chemoradiotherapy [15], [16]. Conventional imaging modalities, including CT and MRI, provide anatomical information but are constrained by reliance on contrast agents, millimeter-scale spatial resolution, and susceptibility to motion artifacts. ^18^F-FDG PET/CT offers quantitative insights into tumor metabolism and has become a standard for staging and response assessment in lymphoma [17], [18], [19], [20]. However, high cost, radiation exposure, and variable prognostic performance in certain indolent subtypes limit its utility for longitudinal monitoring [21].

Photoacoustic imaging (PAI) is an emerging hybrid modality combining optical excitation with acoustic detection, enabling high-resolution visualization of tumor vasculature and oxygenation through spectral unmixing of endogenous chromophores, such as hemoglobin, as well as exogenous probes [11], [22]. Early feasibility studies in breast cancer, melanoma, and metastatic lymph nodes demonstrate its safety and translational potential, particularly for superficial lesions [23], [24], [25], [26], [27]. Preclinical work further shows that PAI can noninvasively track dynamic changes in oxygenation and vascular responses during chemotherapy, vascular-disrupting agents, and photothermal therapies, highlighting its potential as a functional biomarker for treatment monitoring [28], [29], [30], [31], [32], [33]. Nevertheless, its application in NHL, especially for longitudinal response assessment, remains largely unexplored.

Here, we present the first preclinical evaluation of PAI as a non-invasive, longitudinal tool for monitoring treatment responses in NHL. Using a xenograft model, we quantified PAI-derived vascular and oxygenation parameters and examined their concordance with histopathological and serological measures. We further characterized dynamic changes during disease progression and therapy, and performed exploratory analyses linking imaging biomarkers to downstream treatment outcomes.

## 2 Materials and methods

### 2.1 Cell culture

*Jeko‑1* cells were authenticated by STR profiling (Abiowell, China) and confirmed mycoplasma‑free. Cells (passages 3–10) were cultured in RPMI‑1640 medium (Gibco, USA), supplemented with 10% fetal bovine serum (Vivacell, Germany), 1% penicillin/streptomycin, and 1% L‑glutamine at 37 °C in a humidified 5% CO₂ atmosphere.

### 2.2 Animal model

A xenograft mantle cell lymphoma (MCL) model was established using Jeko-1 cells to investigate longitudinal tumor progression and therapeutic response in non-Hodgkin lymphoma (NHL). All animal procedures reported in this study were approved by the Animal Ethics Committee of Fudan University (Approval 20230101) on the ethical use of laboratory animals.

#### 2.2.1 Animal housing and tumor establishment

Female NSG mice (4–5 weeks old; GemPharmatech Co., Ltd., China) were housed under specific pathogen-free (SPF) conditions with a 12 h light/dark cycle, controlled temperature (22 ± 2 °C), and 50–60% relative humidity. After one week of acclimatization, 100 μL of Jeko-1 cell suspension (1 ×10⁷ cells/mL in PBS) was subcutaneously injected into the right axillary region. Tumor volumes were calculated as V = (L × W^2^) / 2. Tumor-bearing mice were enrolled for imaging once tumor diameters reached 5–7 mm; animals with tumors > 15 mm were excluded.

#### 2.2.2 Group allocation and treatment protocol

**Fig. 1:**
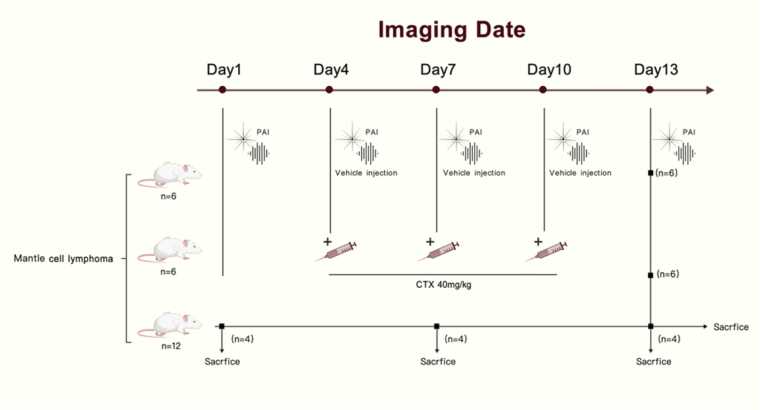
**Experimental design and imaging schedule.** A total of 24 mantle cell lymphoma-bearing mice underwent longitudinal photoacoustic imaging (PAI) on Day 1, Day 4, Day 7, Day 10, and Day 13. Mice were allocated to three groups: vehicle control (n = 6), cyclophosphamide (CTX) treatment (n = 6), and monitoring group (n = 12). Mice in the vehicle control and CTX groups received sterile PBS or CTX (40 mg/kg, i.p.), respectively, on Day 4, Day 7, and Day 10, and were euthanized after the final imaging session on Day 13. In the monitoring group, four mice were euthanized after imaging on Day 1, Day 7, and Day 13 for pathological and serological analyses.

A total of 24 female NOD.Cg-Prkdc^scid Il2rg^tm1Wjl/SzJ (NSG) mice were included and underwent longitudinal PAI at five time points (Day 1, Day 4, Day 7, Day 10, and Day 13). Mice were then randomly assigned to three experimental groups (Fig. 1): Vehicle control (n = 6, sterile PBS, i.p. on Days 4, 7, and 10), Cyclophosphamide treatment (n = 6, CTX, 40 mg/kg i.p. on Days 4, 7, and 10)**,** Time-point monitoring (n = 12). In the monitoring group, four mice were euthanized after PAI on Days 1, 7, and 13 for histopathological analysis.

This study design enabled dynamic monitoring of tumor growth and vascular characteristics over time, evaluation of cyclophosphamide-induced therapeutic effects independent of handling or vehicle interference, and correlation of PAI findings with histopathological features at defined time points.

#### 2.2.3 Photoacoustic imaging system and data processing

**Imaging system and setup:** Multispectral optoacoustic tomography (MSOT) was performed using the **ViewMSOT inVision** (iThera Medical GmbH, Munich, Germany), which is equipped with a 256-element toroidal ring-shaped transducer array operating at 10 Hz. The tunable laser source provided pulsed light at wavelengths ranging from 700 to 875 nm (Fig. S2), and the central transducer frequency was set to 5 MHz.

**Scanning protocol:** All mice were anesthetized with isoflurane (2–2.5% for induction and 1.5–2.0% for maintenance in oxygen) and maintained on a heating pad to ensure physiological stability. They were then positioned in the water-coupled MSOT chamber for 5–10 min under the same preset, temperature-controlled conditions. Sequential transverse MSOT images were acquired with a step size of 0.5 mm, typically beginning at the base of the skull and ending at the lower pole of the kidneys, ensuring complete coverage of the tumor region. Following terminal imaging, mice were euthanized by cervical dislocation, and excised tumors were immediately fixed in 4% paraformaldehyde for 24–48 h before paraffin embedding and sectioning.

**Image reconstruction and spectral unmixing:** Raw MSOT data were first reconstructed. All images included in the analysis then underwent light fluence correction using the built-in function of the viewMSOT software (version 5.1.1.13; iThera Medical GmbH, Munich, Germany). Representative images before and after correction are shown in Supplementary Figs. S7-S8. Multispectral processing was subsequently performed on the corrected images using preset absorption spectra of deoxyhemoglobin (Hb) and oxyhemoglobin (HbO₂), from which MSOT oxygen saturation (mSO₂) and total hemoglobin (HbT) maps were automatically generated.

Regions of interest (ROIs) were manually drawn over tumor regions on reconstructed transverse slices. Tumor localization was determined based on the known right axillary subcutaneous implantation site and longitudinal observation of tumor growth, with reference to corresponding ultrasound (US) and PAI images acquired in the same plane, allowing reliable discrimination of tumor tissue from surrounding non-tumor structures during ROI delineation (Fig. S5). Quantitative values for Hb, HbO₂, HbT and mSO₂ were extracted from these ROIs and exported for further statistical analysis. The primary quantitative analysis was performed on the largest tumor cross-sectional slice at each imaging time point to maintain a consistent plane for longitudinal comparison. As a supplementary assessment, the largest-slice measurements were compared with whole-tumor 3D measurements in three untreated control mice and three CTX-treated mice across all five time points. Whole-tumor mean values of Hb, HbO₂, HbT, and mSO₂ were calculated from ROIs drawn on all tumor-containing transverse slices and paired with the corresponding largest-slice values. Inter-slice heterogeneity of mSO₂ was assessed using the coefficient of variation (CV) across all tumor-containing slices and across the largest slice with adjacent ±3 slices. In addition, qualitative three-dimensional assessment of the whole tumor was performed by reviewing serial transverse MSOT slices across the tumor volume, allowing visualization of the spatial distribution and heterogeneity of functional parameters; representative examples are provided in the Supplementary (Figs. S3-S4).

### 2.3 Pathology and immunohistochemistry

Formalin-fixed, paraffin-embedded (FFPE) xenograft tumors were sectioned at 4 µm. Hematoxylin and eosin (H&E) staining was performed using standard protocols to confirm overall morphology of the xenografts. Immunohistochemistry (IHC) was performed to evaluate tumor proliferation using an anti-Ki67 antibody (Servicebio, GB111499, 1:1000) with HRP–DAB detection and hematoxylin counterstaining. Multiplex immunofluorescence (IF) was performed sequentially for CD31 (Abcam, ab182981, 1:2000), VEGF (Abcam, ab52917, 1:1000), HIF-1α (Abcam, ab308433, 1:1000), and α-SMA (Abcam ab209435, 1:1000) using tyramide signal amplification, with DAPI nuclear counterstain (Fig. S6). Images were acquired using an Olympus fluorescence microscope.

Quantification was performed on five randomly selected non-overlapping fields per tumor at 40 × magnification, and image analysis was conducted in Fiji using uniform thresholds for each marker across all samples. The Ki-67 index, VEGF, and HIF-1α were quantified as positive area fractions. Microvessel area (MVA) was quantified as the CD31-positive area fraction. Microvessel density (MVD) was defined as the number of CD31-positive microvessels per high-power field (HPF). Vessel maturation index (VMI) was defined as the ratio of the α-SMA-covered vascular area to the total CD31-positive vascular area and was calculated as follows:

$$\mathrm{VMI}=\frac{\mathrm{Area}\left(\alpha -\mathrm{SMA}\cap \;\mathrm{CD}31\right)}{\mathrm{Area}\left(\mathrm{CD}31\right)}$$

### 2.4 Serological assays

Serum LDH activity was determined using an automated biochemical analyzer (Mindray, Shenzhen, China). Serum VEGF-A and β_2_-microglobulin (β_2_-MG) concentrations were measured using commercial ELISA kits (Mindray, BPE20260 for VEGF-A; BPE20927 for β_2_-MG) according to the manufacturer’s instructions.

### 2.5 Statistical analysis

All statistical analyses were performed in Python 3.10 (scipy, statsmodels; pingouin when available). Within-group×time effects were evaluated by repeated-measures ANOVA with Greenhouse–Geisser correction when applicable; otherwise, Friedman tests were used. Post hoc pairwise time comparisons were adjusted using the Benjamini–Hochberg FDR. Group × time interactions were examined using linear mixed-effects (LMM) models with time modeled as a categorical factor and a random intercept. Between-group differences at each time point were evaluated using Mann–Whitney *U* test, as appropriate. One-way ANOVA or Kruskal-Wallis assessed temporal trends in pathological and imaging parameters among three sacrificed groups, and post hoc comparisons were performed using Tukey’s HSD test or Holm–Bonferroni correction depending on data distribution. To assess dynamic changes, ΔmSO₂, ΔHb, ΔHbO₂, and slope_mSO₂ (ΔmSO₂/days) were approximated as finite differences over predefined intervals, and their associations with other variables were examined using Pearson correlation analysis. Correlation analyses were treated as exploratory, and P values were adjusted using the Benjamini–Hochberg false discovery rate method. For the supplementary 2D/3D comparison, Pearson correlation analysis and paired scatter plots were used to assess the relationship between the largest-slice and whole-tumor 3D measurements. Descriptive longitudinal curves were plotted as mean ± SD. Inter-slice CVs between whole-tumor 3D slices and the largest slice ±3-slice range were compared using paired tests, as appropriate. Results are reported as mean ± 95% CI where appropriate. A two-sided *P*< 0.05 was considered statistically significant.

## 3 Results

### 3.1 Longitudinal PAI delineates hypoxia evolution in lymphoma

To determine whether PAI can dynamically track vascular and oxygenation changes during lymphoma progression, we established a subcutaneous mantle cell lymphoma model and performed MSOT imaging at five time points in three groups: natural progression monitoring, CTX treatment, and vehicle control. Histological and serological analyses were used to interpret the vascular structural and functional changes reflected by the PAI metrics.

**Fig. 2:**
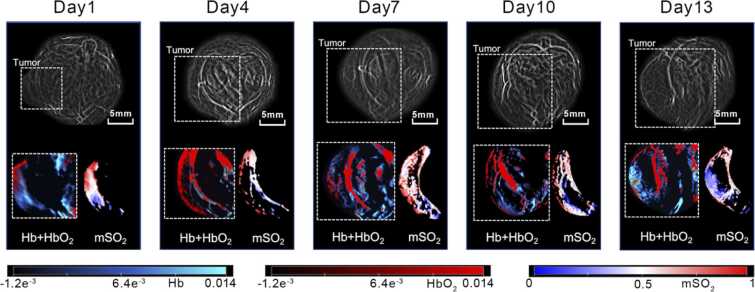
**Representative longitudinal photoacoustic images of the tumor-bearing region.** Images acquired from the same tumor-bearing mouse at Day 1, Day 4, Day 7, Day 10, and Day 13 are shown. The upper panels show overview images of the tumor-bearing region, with dashed boxes indicating the approximate tumor area. Enlarged views of the boxed regions are shown below. The lower left panels show merged Hb and HbO₂ maps, where blue indicates Hb and red indicates HbO₂, and the lower right panels show the corresponding mSO₂ maps. Scale bar = 5 mm.

**Fig. 3:**
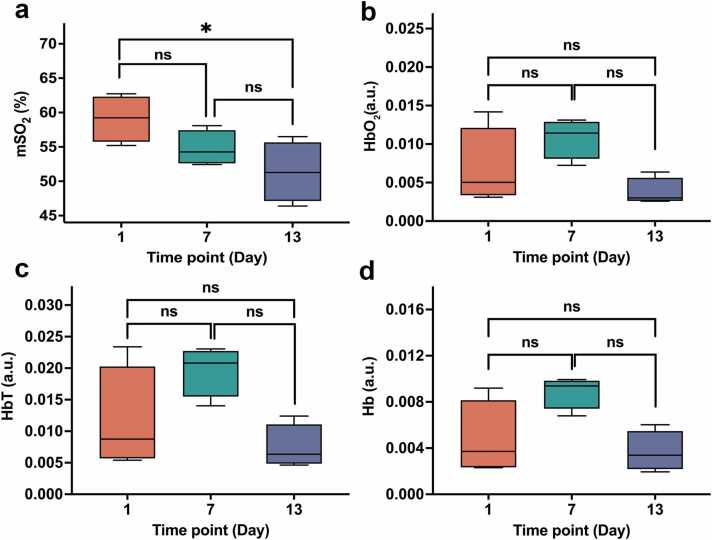
**Longitudinal changes in photoacoustic parameters during tumor progression**. (a) mSO₂, (b) HbO₂, (c) HbT, and (d) Hb. mSO₂ showed a downward trend over time and was significantly lower at Day 13 than at Day 1. Hb and HbO₂ also tended to decrease from Day 7 to Day 13, although these pairwise differences did not reach statistical significance after Holm correction (both adjusted *P* = 0.0558). No significant pairwise differences were observed for HbT, and the remaining comparisons for Hb and HbO₂ were also not statistically significant. Data are presented as boxplots, with the center line indicating the median, the box indicating the interquartile range, and whiskers indicating the minimum to maximum. Overall differences among time points were assessed using the Kruskal–Wallis test, followed by Dunn’s post hoc test with Holm correction for multiple comparisons. *P < 0.05; ns, not significant.

**Fig. 4:**
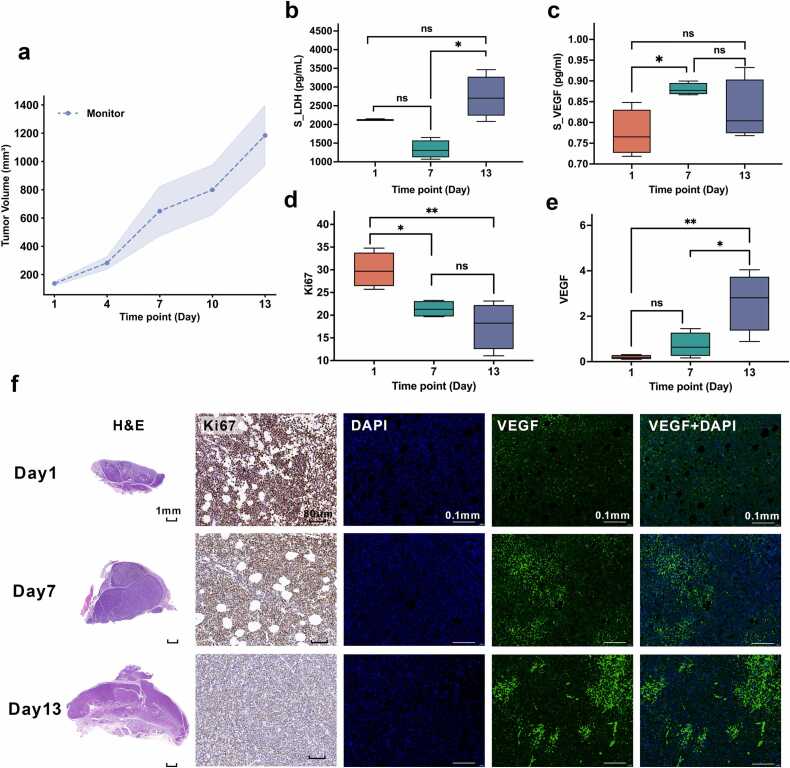
Longitudinal changes in tumor growth, serum markers, and pathological indicators in the monitoring group. (a) Tumor volume curve of the monitoring group from Day 1 to Day 13. The dashed line indicates the mean, and the shaded area indicates the 95% CI. (b–e) Boxplots of serum lactate dehydrogenase (S_LDH), serum vascular endothelial growth factor (S_VEGF), Ki-67-positive area fraction, and VEGF-positive area fraction at Day 1, Day 7, and Day 13. During tumor progression, S_LDH and VEGF-positive area fraction tended to increase, whereas Ki-67-positive area fraction decreased at later time points. In contrast, S_VEGF showed a transient increase at Day 7. Significant pairwise differences are indicated in the panels; all other comparisons were not statistically significant. (f) Representative HE and Ki-67 staining images, together with DAPI, VEGF, and merged VEGF+DAPI fluorescence images, at Day 1, Day 7, and Day 13. In the boxplots, the center line indicates the median, the box indicates the interquartile range, and the whiskers indicate the minimum to maximum. Overall differences among time points were assessed using the Kruskal–Wallis test, followed by Dunn’s post hoc test with Holm correction for multiple comparisons. Scale bars: HE, 1 mm; Ki-67, 80 μm; immunofluorescence images, 0.1 mm.*P < 0.05; **P < 0.01; ns, not significant.

In the natural-progression group, longitudinal MSOT visualized tumor growth (Fig. 4a) and the progressive expansion of hypoxic regions (Fig. 3a). Early-stage tumors exhibited relatively uniform oxygenation, whereas late-stage tumors developed enlarged low-oxygen zones with an overall shift toward lower oxygen saturation (Fig. 2).

Quantitatively, mSO₂ decreased during natural progression (Fig. 3a). Hemoglobin-derived signals, such as Hb (Fig. 3d), HbO₂ (Fig. 3b), and HbT (Fig. 3c), showed a slight and transient increase at mid-stage (Day 7) followed by a reduction by Day 13.

Endpoint analyses supported these imaging findings: as S_LDH increased (Fig. 4b), intratumoral VEGF expression progressively increased (Fig. 4e), accompanied by a rise in S_VEGF (Fig. 4c). In contrast, Ki-67 expression declined over time (Fig. 4d). Representative H&E, Ki-67, and VEGF staining images are shown in Fig. 4f. Collectively, these findings demonstrated a characteristic natural-progression pattern marked by tumor expansion, enhanced pro-angiogenic signaling, a progressively hypoxic microenvironment, and reduced proliferative activity, thereby providing a biological reference for treatment-response evaluation.

### 3.2 PAI identifies early oxygenation shifts preceding volumetric treatment response

**Fig. 5:**
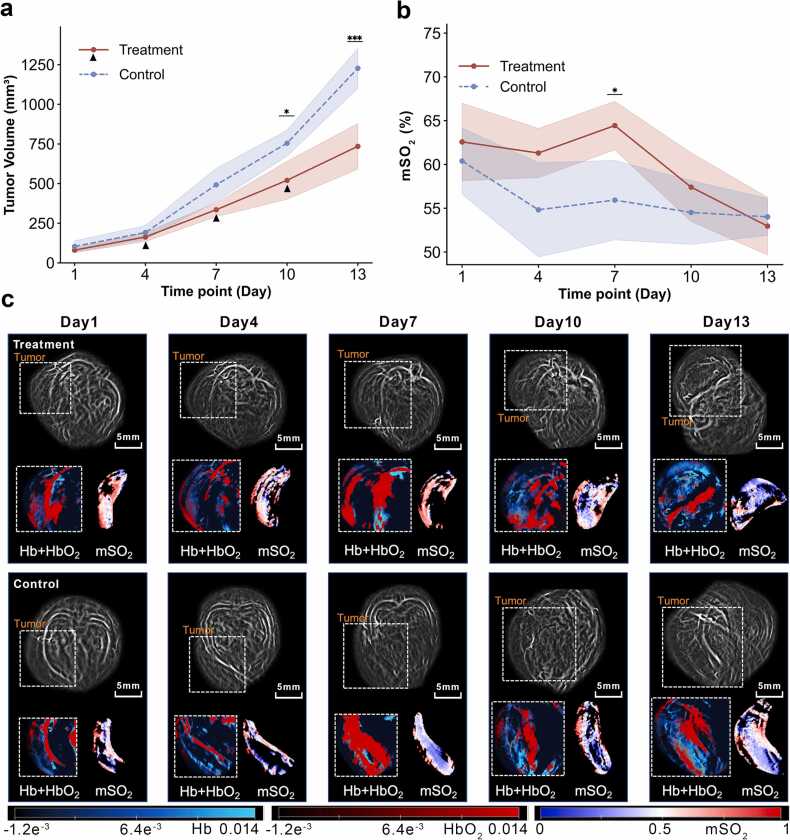
Longitudinal changes in tumor volume and mSO₂ in the treatment and control groups, with representative photoacoustic images at each imaging time point. (a) Tumor volume curves of the treatment and control groups from Day 1 to Day 13. Tumor volume increased over time in both groups, but growth was slower in the treatment group, with significant between-group differences in tumor volume at Day 10 and Day 13. (b) Longitudinal changes in mSO₂ in the treatment and control groups over the same period. The treatment group showed a transient increase in mSO₂ at the intermediate stage, followed by a decline at later time points, whereas the control group exhibited an overall downward trend. (c) Representative photoacoustic images of the treatment and control groups at Day 1, Day 4, Day 7, Day 10, and Day 13. The first and second rows show serial images acquired from the same tumor-bearing mouse in the treatment and control groups, respectively, across the five imaging time points. Within each time-point panel, the upper image shows an overview of the tumor-bearing region, with dashed boxes indicating the approximate tumor area, and the lower image shows an enlarged view of the boxed region. Merged Hb and HbO₂ maps are shown on the left, where blue indicates Hb and red indicates HbO₂, and the corresponding mSO₂ maps are shown on the right. In panels (a) and (b), solid and dashed lines represent the treatment and control groups, respectively, and shaded areas indicate the 95% CI. Scale bar = 5 mm. *P < 0.05; ***P < 0.001.

Tumor volumes increased in both CTX and control groups, but diverged after treatment initiation at Day 4, with significantly slower growth in CTX-treated tumors at Day 10 (*P* < 0.05) and Day 13 (*P* < 0.001), supported by a significant time × group interaction (Fig. 5a), and individual tumor growth curves are also displayed (Fig. S1).

**Fig. 6:**
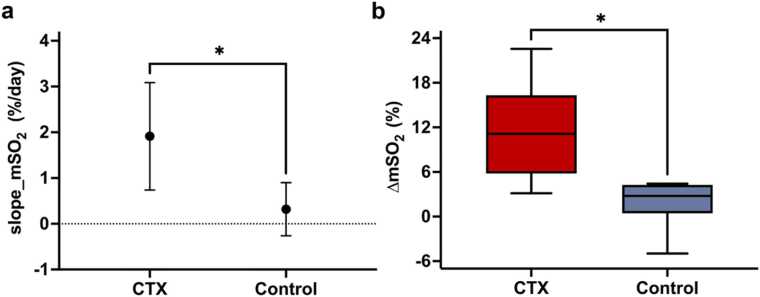
**Comparison of the magnitude of late-stage mSO₂ decline between the CTX and control groups.** (a) Comparison of the slope of mSO₂ change from Day 7 to Day 13 between the CTX and control groups. The CTX group showed a steeper decline in mSO₂ than the control group. The dotted horizontal line indicates zero change. (b) Boxplot of ΔmSO₂ in the CTX and control groups. ΔmSO₂ represents the change in mSO₂ from Day 7 to Day 13, calculated as Day 7 minus Day 13, with higher values indicating a greater decrease in mSO₂ over time. Compared with the control group, the CTX group showed a larger decline in mSO₂. In the boxplot, the center line indicates the median, the box indicates the interquartile range, and the whiskers indicate the minimum to maximum. Between-group differences were assessed using the Mann–Whitney *U* test.

PAI revealed a markedly different oxygenation trajectory under CTX therapy (Fig. 5c). Baseline tumor characteristics were comparable between groups. At Day 7, mSO₂ was significantly higher in the CTX group than in controls [64.66 (62.90, 67.13) vs 57.54 (56.83, 58.89), *P* = 0.0087]. This early separation was not maintained. In addition, Hb and HbT differed significantly between groups at the mid-treatment stage, with lower values in the CTX group at Day 4 and Day 7. From Day 7 to Day 13, mSO₂ declined in both groups, but the decrease was greater in the CTX group. Consistently, slope_mSO₂ [1.85 (1.25, 2.29) vs 0.46 (0.39, 0.65), *P* = 0.0173] and ΔmSO₂ [11.12 (7.52, 13.70) vs 2.78 (2.33, 3.90), *P* = 0.0173] were both higher in the CTX group. By Day 13, absolute mSO₂ values were similar between groups, whereas the longitudinal indices still captured clear differences in oxygenation dynamics (Figs. 6a-6b).

**Fig. 7:**
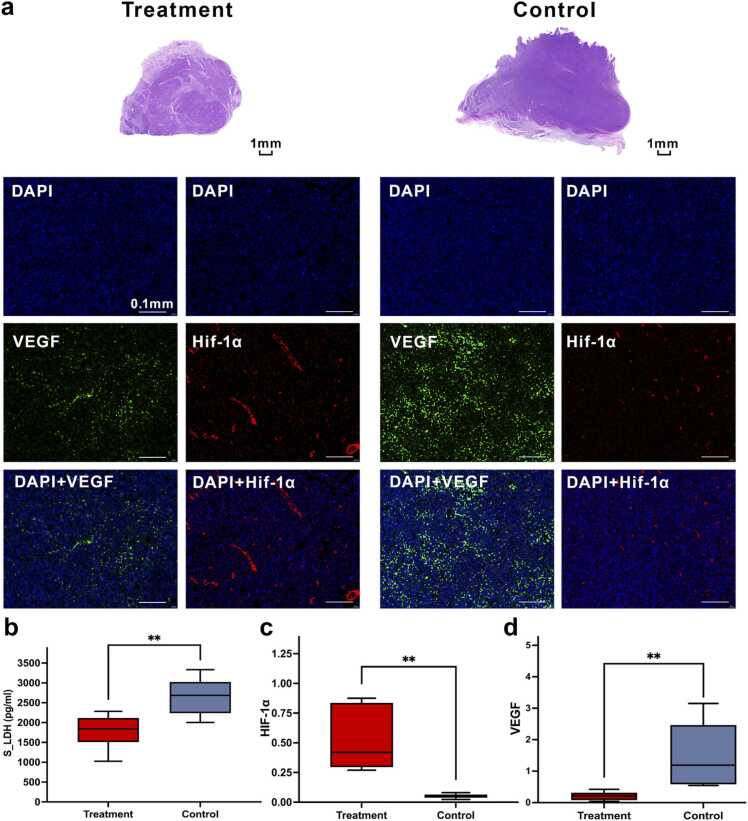
Endpoint histopathological, immunofluorescence, and serological differences between the treatment and control groups. (a) Representative HE, DAPI, VEGF, HIF-1α, and merged immunofluorescence images from the treatment and control groups at the endpoint. Compared with the treatment group, the control group showed stronger VEGF fluorescence, whereas HIF-1α fluorescence was more prominent in the treatment group. (b–d) Boxplots of serum lactate dehydrogenase (S_LDH), HIF-1α-positive area fraction, and VEGF-positive area fraction in the treatment and control groups. S_LDH and VEGF-positive area fraction were higher in the control group, whereas HIF-1α-positive area fraction was higher in the treatment group. In the boxplots, the center line indicates the median, the box indicates the interquartile range, and the whiskers indicate the minimum to maximum. Between-group differences were assessed using the Mann–Whitney *U* test. Scale bars: HE, 1 mm; immunofluorescence images, 0.1 mm. **P < 0.01.

Pathological and serologic analyses at the endpoint further supported the imaging findings: intratumoral VEGF-positive area (Fig. 7d) and serum LDH (Fig. 7b) were lower in the CTX group than in controls, consistent with cytotoxic/anti-proliferative treatment effects (*P* < 0.05). The HIF-1α positive area fraction was higher in the CTX group at the endpoint (Fig. 7c). Serum VEGF and β2-microglobulin showed no significant group differences.

### 3.3 CTX promotes hypoxia-associated microvascular immaturity

**Fig. 8:**
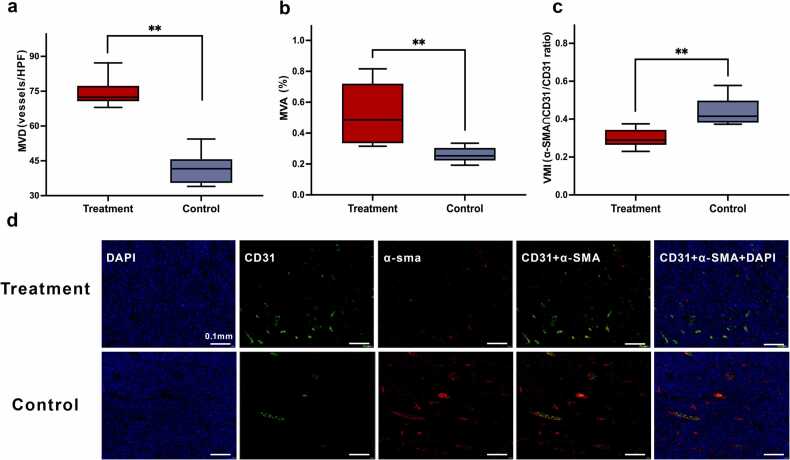
Differences in microvascular parameters and representative immunofluorescence images between the treatment and control groups at the endpoint. (a–c) Boxplots of microvessel density (MVD), microvessel area fraction (MVA), and vascular maturity index (VMI) in the treatment and control groups. Compared with the control group, the treatment group showed higher MVD and MVA, but lower VMI. (d) Representative DAPI, CD31, α-SMA, merged CD31 + α-SMA, and merged CD31 + α-SMA+DAPI immunofluorescence images from the treatment and control groups at the endpoint. CD31 is shown in green, α-SMA in red, and DAPI in blue. Compared with the control group, the treatment group showed greater CD31-positive microvessel density but relatively reduced α-SMA coverage, consistent with increased vessel abundance and lower vascular maturity. In the boxplots, the center line indicates the median, the box indicates the interquartile range, and the whiskers indicate the minimum to maximum. Between-group differences were assessed using the Mann–Whitney *U* test. Scale bar = 0.1 mm, **P < 0.01.

Despite tumor growth reduced with mSO₂ continued to decline, there was no significant decrease in Hb-based PAI signals relative to controls during the late treatment phase. To clarify whether this reflected vascular normalization or aberrant remodeling, endpoint microvascular parameters were evaluated (Fig. 8). CTX-treated tumors exhibited a significantly increased MVD (Fig. 8a) and MVA (Fig. 8b) but markedly reduced VMI (Fig. 8c), along with higher HIF-1α expression at the endpoint (Fig. 7c). These findings indicate that CTX was associated with an immature, structurally unstable vascular phenotype characterized by increased vessel number but reduced function, thereby maintaining hypoxia.

### 3.4 Therapy reconfigures oxygenation–vascular architecture relationships

**Fig. 9:**
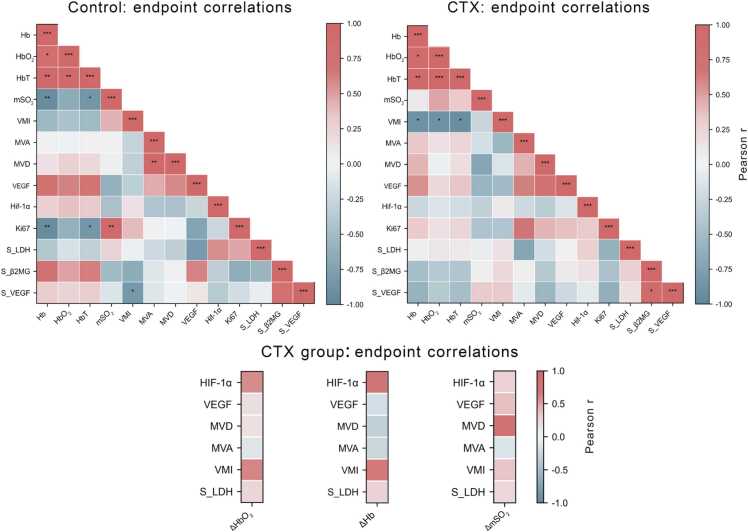
Correlation heatmaps of PAI parameters, pathological markers and serological biomarkers. (a, b) Endpoint Pearson correlation heatmaps for the control group (a) and CTX-treated group (b), showing the relationships among PAI parameters, vascular indices, hypoxia- and angiogenesis-related markers, and serum biomarkers. (c) Pearson correlation heatmap showing the associations between imaging-derived dynamic metrics (ΔHbO₂, ΔHb, and ΔmSO₂) over the treatment interval and selected endpoint pathological and serological measures in the CTX group; Δ indicates the difference calculated as Day 7 minus Day 13. Red indicates positive correlations and blue indicates negative correlations, with color intensity corresponding to Pearson’s correlation coefficient (*r*). Asterisks indicate nominal significance before multiple-comparison correction (*P < 0.05, **P < 0.01, ***P < 0.001). Correlation analyses were exploratory. After Benjamini–Hochberg correction, statistically significant associations were mainly retained among PAI-derived parameters, whereas correlations involving pathological or serological variables were no longer statistically significant.

Endpoint correlation matrices revealed that control tumors retained a relatively coherent coupling among vascular density, vessel maturity, and oxygenation. In contrast, CTX treatment disrupted or reconfigured many of these associations, particularly with respect to tumor vascular remodeling (Fig. 9a, Fig. 9b). From Day 7 to Day 13, the greater late-stage decline in mSO₂ in the CTX group, reflected by a higher ΔmSO₂, is consistent with more severe hypoxia, suggesting that hypoxia-related angiogenic responses and structural vascular remodeling may occur in parallel. Nominally, Hb, HbO₂, and HbT showed inverse correlations with VMI, and mSO₂ showed the same directionality, although these associations did not remain statistically significant after multiple-comparison correction. Meanwhile, dynamic changes in ΔHb and ΔHbO₂ showed concordant trends with VMI (Fig. 9c). Notably, compared with single-time-point measurements, dynamic PAI metrics may provide a more sensitive readout of therapy-induced alterations in vascular function.

As a supplementary analysis, we compared the largest-slice measurements with whole-tumor 3D measurements in representative control and CTX-treated mice. Hb, HbO₂, HbT, and mSO₂ showed close correlations between the two approaches, although the values were not identical (Fig. S9). The main longitudinal mSO₂ pattern, including the transient increase around Day 7 and subsequent decline in the CTX-treated group, was also observed in the whole-tumor 3D analysis (Fig. S10). Slice-by-slice mSO₂ analysis further showed a higher CV across whole-tumor 3D slices than within the largest slice ±3-slice range, suggesting greater inter-slice heterogeneity when the entire tumor volume was included (Fig. S11 and Table S1).

## 4 Discussion

Assessment of treatment response in lymphoma remains dominated by structural imaging and FDG–PET criteria [34], [35], and noninvasive biomarkers that dynamically report intratumoral vascular and oxygenation changes are scarce. This study extends optoacoustic therapy monitoring from solid tumors to lymphoma and provides initial evidence that PAI can quantify CTX–induced mSO₂ dynamic effects in a mantle cell lymphoma xenograft model. By directly contrasting a natural-progression group with CTX-treated tumors, we observed divergent mSO₂ trajectories: a pronounced decline in mSO₂ superimposed on the progressive hypoxia observed in untreated controls.

To link the PAI readouts with histological and serological changes, we used a staged-sacrifice group to capture the natural history of tumor progression. In that group, increasing tumor volume was accompanied by a steady fall in mSO₂, consistent with worsening functional hypoxia. Tissue markers shifted toward a hypoxic, pro-angiogenic yet proliferation-suppressed phenotype, with higher VEGF and S_LDH and lower Ki-67. These observations are consistent with previous PAI/OE-MSOT and direct pO₂ studies showing that tumor growth is typically accompanied by increasing hypoxia [36], [37], supporting mSO₂ as a plausible noninvasive hypoxia readout and providing a reference against which CTX-induced deviations can be interpreted.

When we examined CTX effects longitudinally, the most discriminative signal emerged in the mid-to-late treatment window. mSO₂ in CTX-treated tumors showed a transient increase at the early stage after treatment, but this change was not sustained and was followed by a steeper decline at later time points. Although Hb, HbO₂, and HbT also changed during treatment, the oxygenation trajectory remained the most distinctive feature. This pattern suggests preserved or increased intratumoral blood content, yet without a corresponding improvement in oxygen delivery, consistent with non-productive perfusion rather than vascular normalization [38]. The transient early rise in mSO₂ may reflect short-lived relative reoxygenation after partial tumor cell killing, whereas the subsequent decline suggests that the treatment effect was accompanied by further remodeling of the tumor microenvironment. Mechanistically, this may involve endothelial injury, pruning of functional microvessels, and cycles of tumor cell kill and repopulation reported for CTX-containing regimens, which can deepen or prolong tumor hypoxia [39], [40], [41].

Endpoint vascular pathology mirrored the functional imaging: CTX-treated tumors showed increased CD31-defined microvessel density and area, accompanied by reduced α-SMA coverage, markedly immature, irregular vessels, persistent HIF-1α, and low mSO₂ [41]. This pattern contrasts with the canonical “vascular normalization” observed after some anti-VEGF or radiotherapy regimens, in which vessel density often decreases while maturation and perfusion improve transiently [32], [42]. Instead, our data support a hypoxia-driven, non-productive remodeling process in which CTX increases vessel number but compromises vessel function.

The serological findings were consistent with this interpretation. Intratumoral VEGF was reduced with CTX, likely reflecting reduced viable tumor mass and biosynthetic capacity rather than selective VEGF pathway blockade; circulating s-VEGF did not show a corresponding fall, consistent with the understood noisiness of circulating VEGF (derived from tumor cells, stroma, platelets, and inflammation) and its weak coupling to local HIF-1α–VEGF signalling [30], [43]. By contrast, serum LDH was lower in CTX-treated mice, aligning with reduced tumor burden and metabolic activity (glycolysis) and supporting a genuine cytoreductive effect [44], [45]. Overall, these data favor an interpretation in which the late mSO₂ decline reflects the functional status of newly formed, structurally immature vessels rather than a simple reduction in cellular oxygen demand.

Placing our results alongside prior PAI/OE-MSOT therapy-monitoring studies reveals both similarities and important distinctions. The natural decline in mSO₂ we observed aligns with reports in prostate, breast, and lymphoma models [11], [23], [29], [31], [46], [47], [48], indicating that PAI-derived mSO₂ can serve as a feasible noninvasive measure of tumor oxygenation status. Notably, CTX treatment did not produce a sustained improvement in oxygenation in our model. The later decline, combined with increased MVD/MVA yet reduced VMI, suggests functionally inadequate vascular remodeling. Conversely, previous studies using targeted therapeutic strategies, such as anti-angiogenic therapies (including anti-VEGF monoclonal antibodies) and certain radiotherapy regimens, have reported increased mSO₂, reduced vessel density, and temporary enhancements in perfusion and oxygenation within specific dose and timing conditions (a putative “normalization window”) [30], [42], [43]. This underscores that oxygenation trends should be interpreted considering the therapeutic mechanisms and vascular phenotype. In this study, dynamic monitoring of mSO₂ effectively detected treatment-induced changes in the tumor growth microenvironment before volumetric differences appeared, and further revealed persistent hypoxia linked to functional vascular changes during the mid-to-late treatment phase.

This study has several limitations. First, the sample size was relatively small, which may have limited statistical power and increased the risk of false-negative findings in some subgroup and correlation analyses. Second, the experiments were performed in NSG xenograft models, and histologic and serologic assessments in the treatment cohort were obtained only at the endpoint; therefore, mechanistic interpretation of individual PAI changes remains preliminary. Third, only one CTX dosing regimen was evaluated, and different doses or schedules might lead to different vascular and oxygenation responses. In addition, the primary quantitative analysis was based on the largest cross-sectional slice rather than full volumetric quantification. Although supplementary 2D/3D comparisons showed broadly consistent longitudinal trends, whole-tumor 3D analysis revealed greater inter-slice variation, indicating that the largest-slice-based analysis may not fully capture intratumoral spatial heterogeneity. Larger studies with whole-tumor 3D quantification are needed to further validate this approach.

## 5 Conclusion

Longitudinal PAI captured progressive hypoxia during lymphoma growth and revealed a markedly steeper, CTX-associated decline in mSO₂, with early oxygenation alterations detectable before robust volumetric treatment differences became evident. Our findings indicate that CTX does not induce classical vascular normalization in lymphoma, but instead is associated with a hypoxia-driven, non-productive remodeling process, yielding intratumoral vessels that are structurally more abundant yet functionally compromised. These results surpport dynamic oxygenation trajectories as a promising noninvasive biomarker for treatment-response evaluation and may help inform future studies on therapeutic scheduling in NHL, particularly in combination-therapy settings.

## CRediT authorship contribution statement

**Lin Chen:** Writing – review & editing, Supervision, Resources, Project administration, Funding acquisition, Conceptualization. **Ting Feng:** Writing – review & editing, Supervision, Resources, Project administration, Funding acquisition, Conceptualization. **Dean Ta:** Writing – review & editing, Supervision, Resources, Conceptualization. **Yan Huang:** Visualization, Software, Formal analysis. **Qianping Mao:** Visualization, Software, Formal analysis. **Jie Mi:** Visualization, Software, Formal analysis. **Ying Fan:** Writing – review & editing, Visualization, Data curation, Conceptualization. **Jing Shi:** Writing – original draft, Validation, Software, Formal analysis, Data curation.

## Funding

This work was supported by a grant from the Medical-Engineering Cross Project of Fudan University (Project No. YG2023-16, Chen L).

## Declaration of Competing Interest

The authors declare that they have no known competing financial interests or personal relationships that could have appeared to influence the work reported in this paper.

## Data Availability

Data will be made available upon reasonable request.

## Footnotes

Appendix A Supplementary data associated with this article can be found in the online version at doi:10.1016/j.pacs.2026.100835.
