# Supplementary material for: Quantitative longitudinal photoacoustic imaging of vascular oxygenation for therapy response monitoring in Non-Hodgkin lymphoma
Source: Photoacoustics. 2026 May 13;50:100835. doi: 10.1016/j.pacs.2026.100835 (PMC13213287; doi:10.1016/j.pacs.2026.100835)
Supplement: Supplementary file 1 — Supplementary material [file mmc1.docx]

**Supplementary Figure**


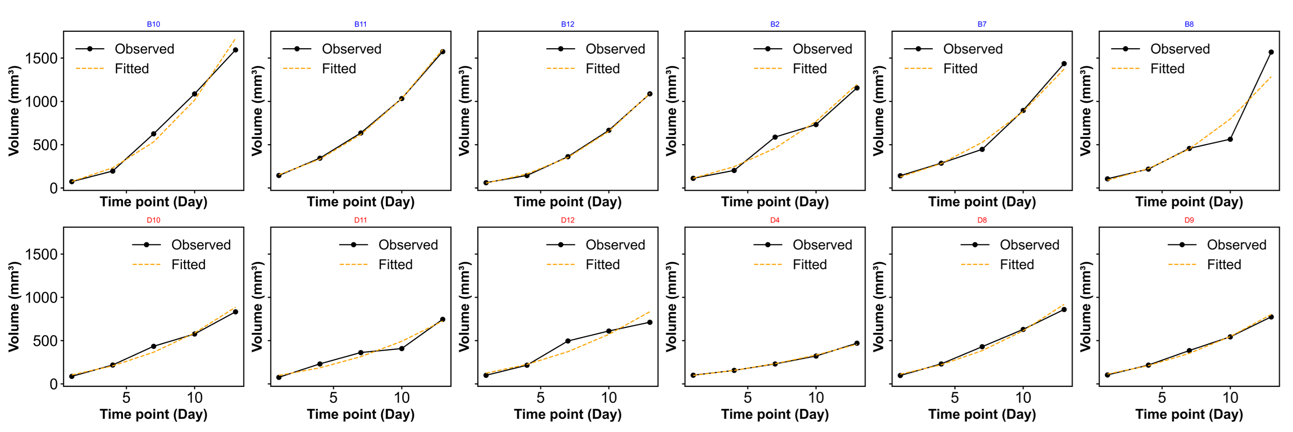


**Fig. S1. Observed and fitted tumor growth curves for individual mice.** Each panel shows the longitudinal tumor volume measurements of one mouse over time. Black solid lines with points indicate the observed values, and orange dashed lines indicate the fitted values. Tumor volume is plotted against imaging time from Day 1 to Day 13.


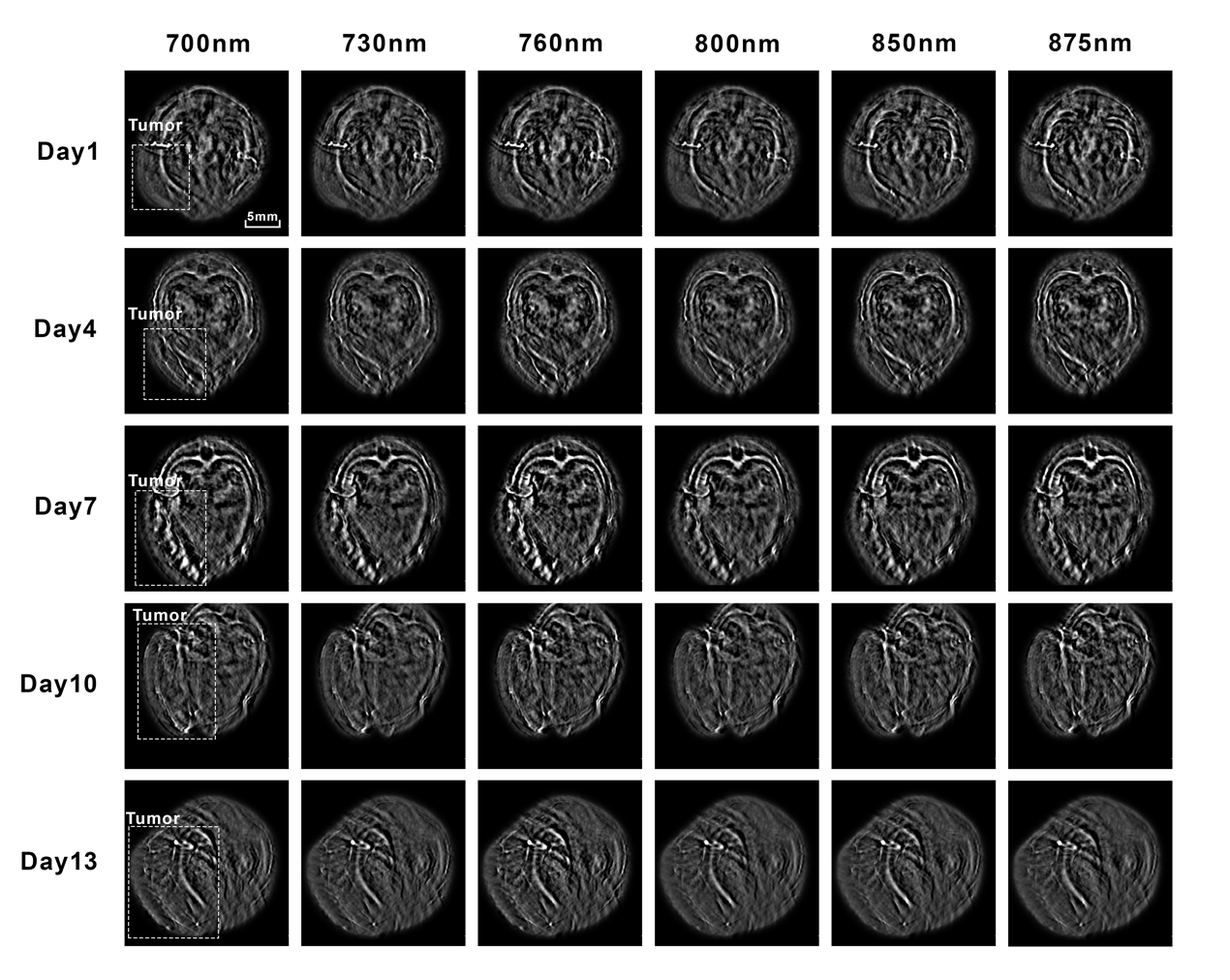


**Fig. S2. Representative raw multi-wavelength photoacoustic images used for hemodynamic analysis.** Cross-sectional photoacoustic images of the same tumor-bearing mouse were acquired longitudinally on Days 1, 4, 7, 10, and 13 at 700, 730, 760, 800, 850, and 875 nm before spectral unmixing to generate the corresponding hemodynamic parameter maps. The dashed boxes indicate the tumor region, and the arrows denote the approximate tumor location. Scale bar = 5 mm.


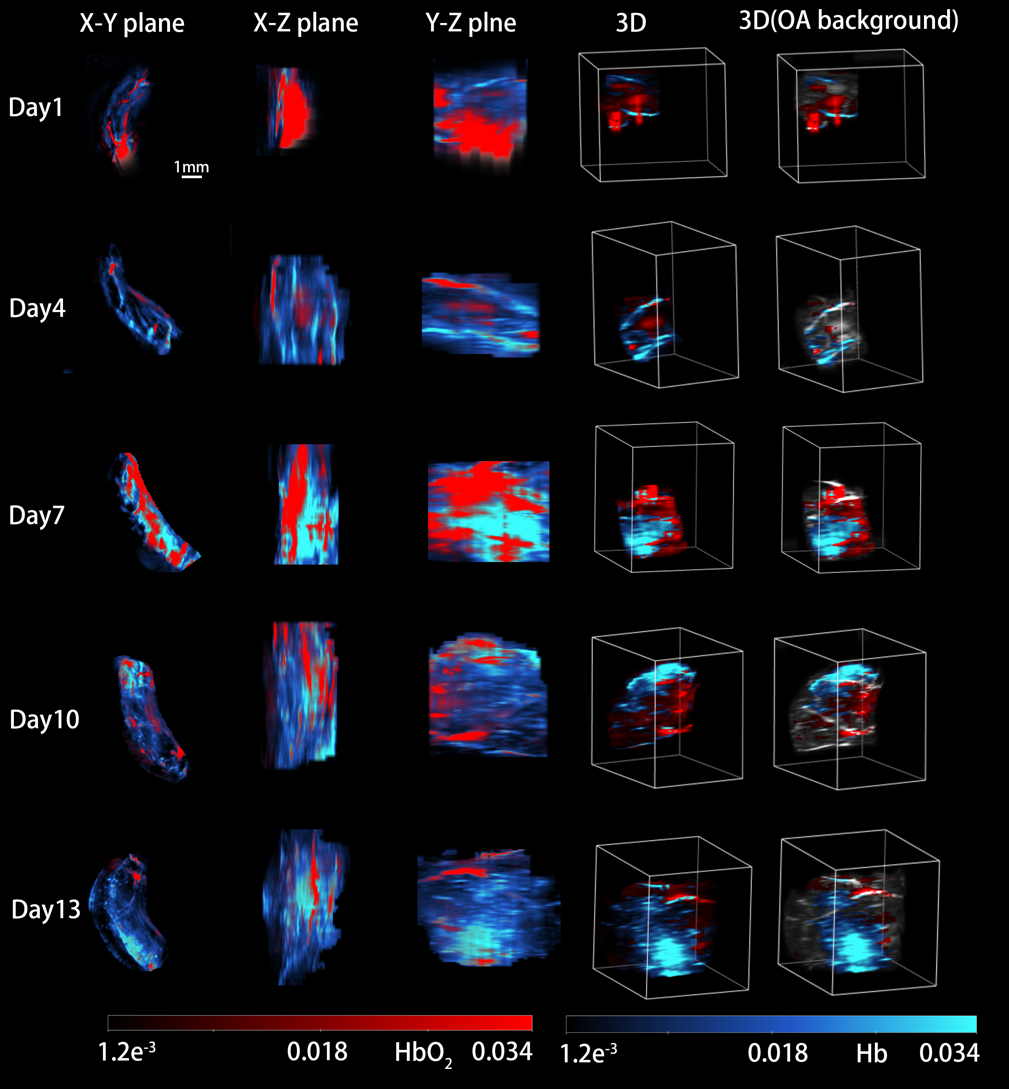


**Fig. S3. Representative three-dimensional photoacoustic reconstructions of a tumor from a mouse in the control group**. For each time point (from Day 1 to Day 13), X-Y, X-Z, and Y-Z plane views, 3D renderings, and 3D renderings overlaid on the PAI background are shown. Red indicates HbO₂ signal, and blue indicates Hb signal.As the tumor progressed, the spatial distribution of HbO₂ and Hb underwent continuous remodeling, with relatively increased Hb and reduced HbO₂ signals at later time points, suggesting progressively aggravated tumor hypoxia.


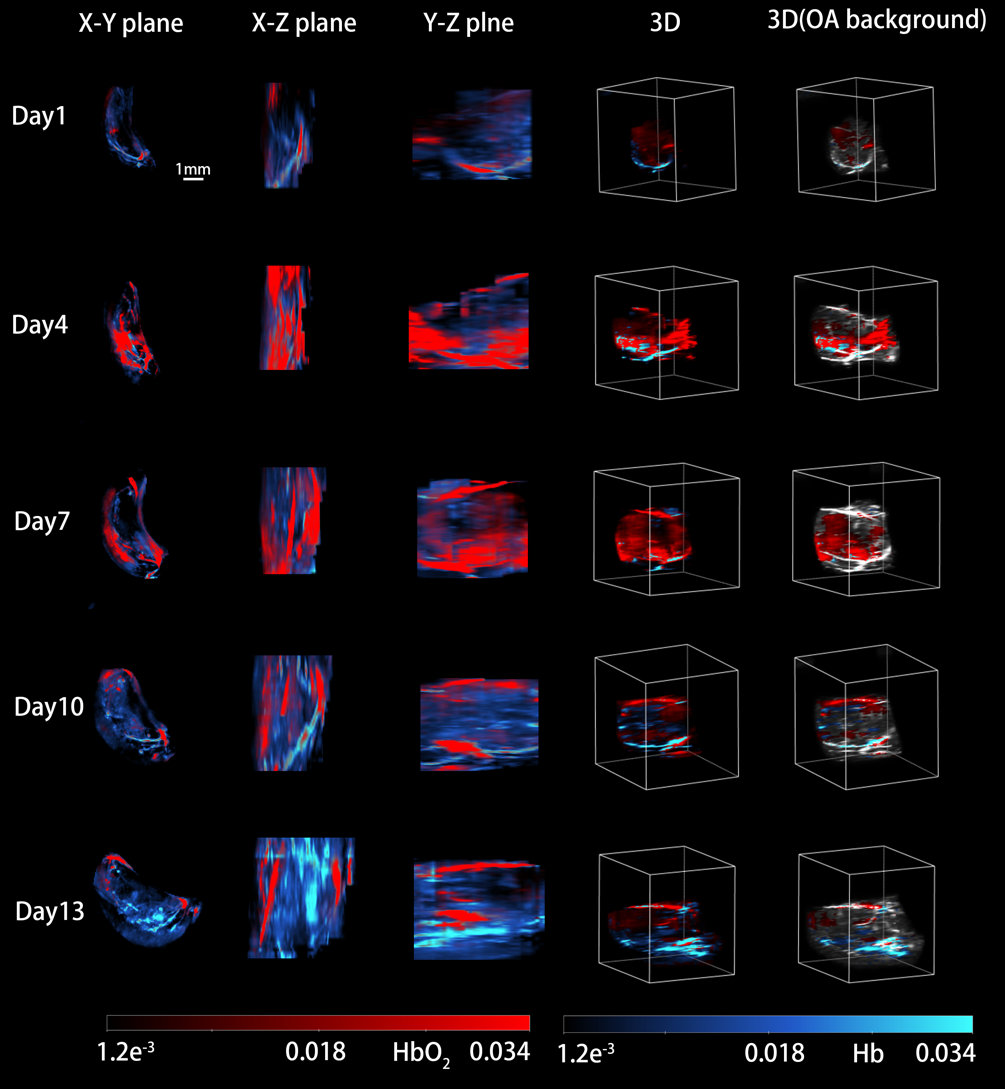


**Fig. S4. Representative three-dimensional photoacoustic reconstructions of a tumor from a mouse in the treatment group**. For each time point (from Day 1 to Day 13), X-Y, X-Z, and Y-Z plane views, 3D renderings, and 3D renderings overlaid on the PAI background are shown. Red indicates HbO₂ signal, and blue indicates Hb signal. A transient increase in HbO₂ signal was observed in the early stage after treatment, suggesting a temporary improvement in oxygenation; however, at later time points, Hb increased, while HbO₂ decreased, indicating a subsequent decline in oxygenation.

**
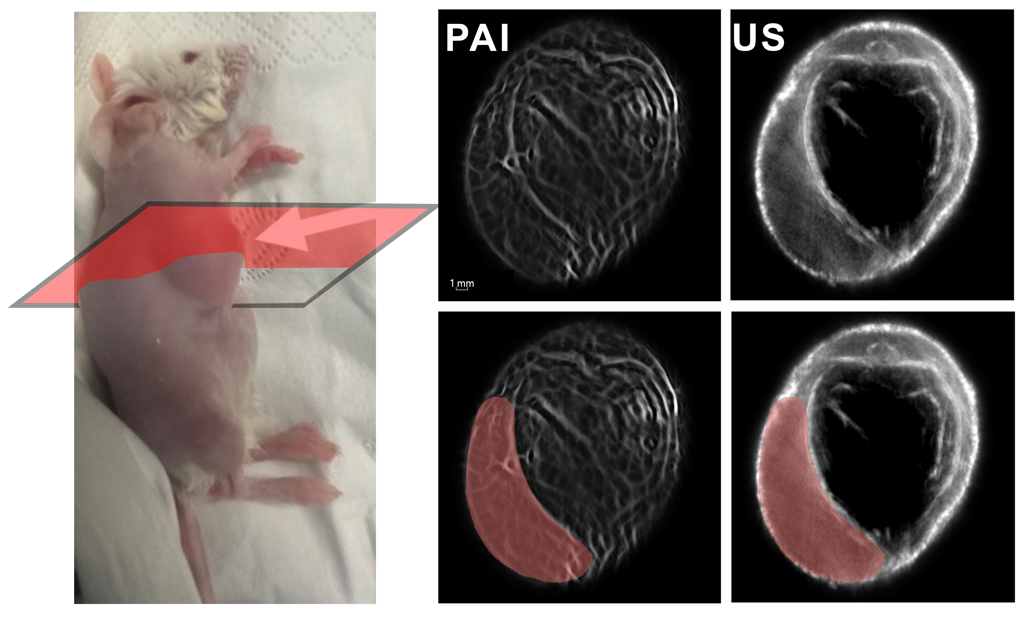
**

**Fig. S5. Schematic illustration of tumor localization and ROI delineation.** Tumor ROIs were delineated by integrating the known implantation site on the body surface (right axillary subcutaneous region) with the corresponding cross-sectional PAI and US images. The left panel shows the body-surface tumor location in a tumor-bearing mouse, with the arrow indicating the right axillary subcutaneous xenograft area. The right panel shows representative cross-sectional PAI and US images at the corresponding level, and the lower row illustrates the manually delineated tumor ROIs based on the original images.


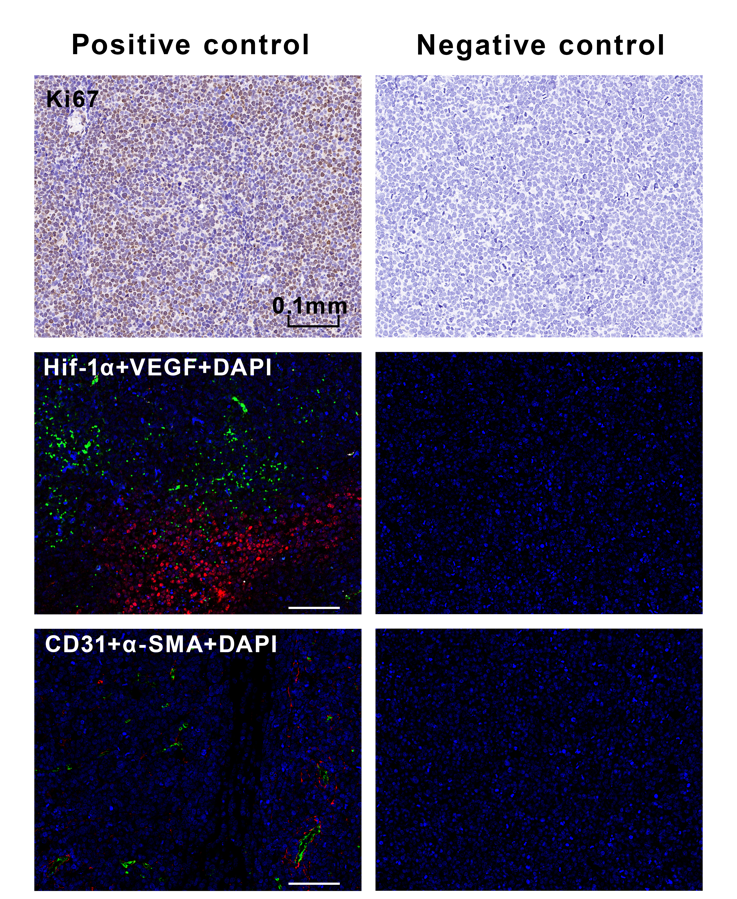


**Fig. S6. Positive and negative controls for histological staining.** Representative positive and negative control images for Ki-67 immunohistochemistry, HIF-1α/VEGF immunofluorescence, and CD31/α-SMA immunofluorescence are shown. The left column shows the positive controls, and the right column shows the corresponding negative controls. In the negative controls, no obvious specific positive staining was observed, supporting the specificity of the staining procedure.


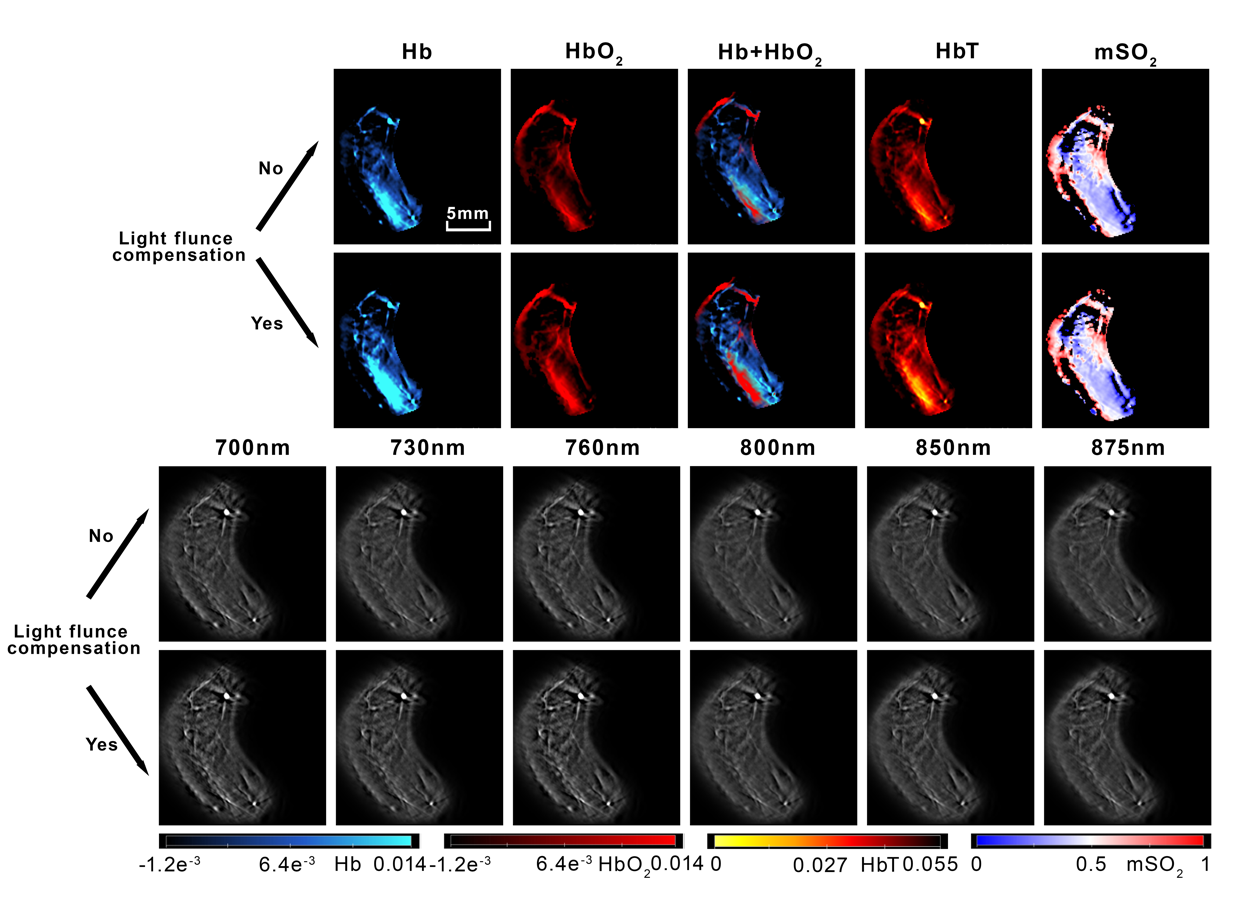


**Fig. S7.** **Representative comparison of photoacoustic images and derived hemodynamic parameter maps with and without light fluence compensation.** The lower panels show representative multi-wavelength photoacoustic images acquired at 700, 730, 760, 800, 850, and 875 nm, reconstructed without (No) or with (Yes) light fluence compensation. The upper panels show the corresponding hemodynamic parameter maps derived from spectral unmixing, including Hb, HbO₂, merged Hb/HbO₂, HbT, and mSO₂. The figure illustrates the visual influence of light fluence compensation on both raw multi-wavelength images and the resulting quantitative hemodynamic maps.


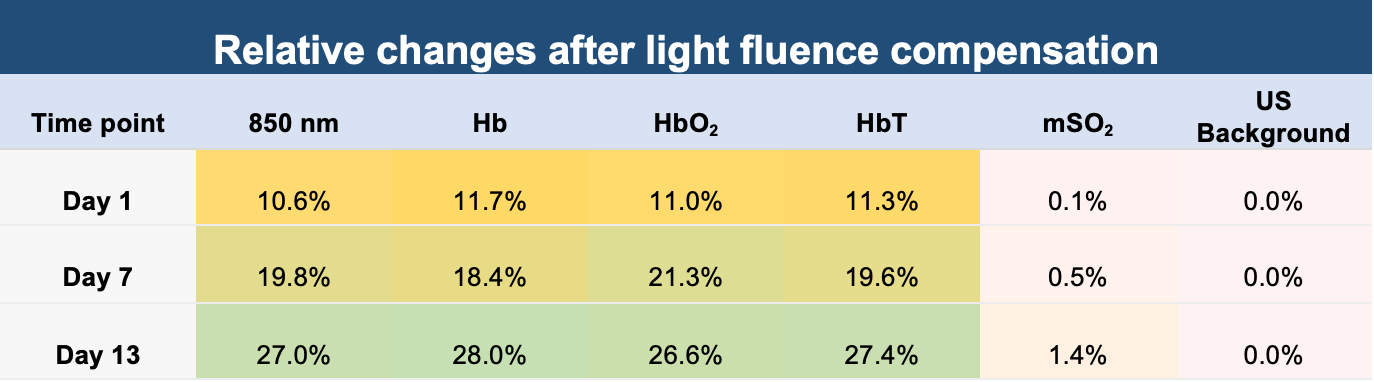


**Fig. S8. Relative changes in quantitative parameters after light fluence compensation.** Heatmap showing the percentage change in quantitative parameters after light fluence compensation in the same tumor from one mouse at three longitudinal time points (Days 1, 7, and 13), calculated as (compensated − uncompensated) / uncompensated × 100%. Light fluence compensation increased the signal intensity-related parameters, including the 850 nm signal, Hb, HbO₂, and HbT, whereas its effect on mSO₂ was minimal and the US background remained unchanged.


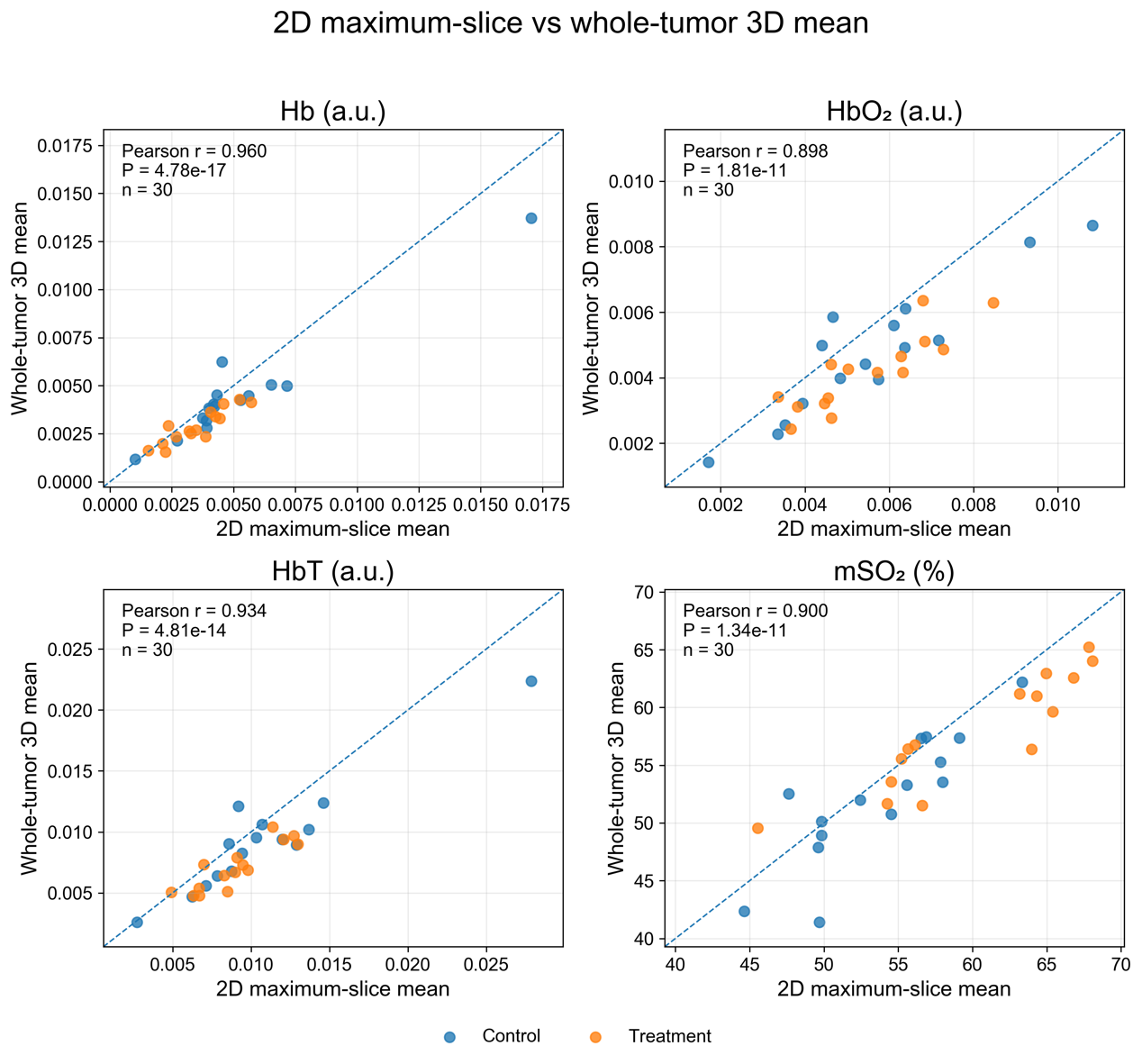


**Fig. S9. Relationship between 2D maximum-slice and whole-tumor 3D measurements of photoacoustic parameters.** For each mouse-time point, the mean value obtained from the 2D maximum cross-sectional slice was paired with the corresponding whole-tumor 3D mean value. Scatter plots are shown for Hb, HbO₂, HbT, and mSO₂. Blue and orange points indicate control and CTX-treated mice, respectively. The dashed line represents the identity line. Pearson correlation coefficients, P values, and sample size are indicated in each panel. The two approaches showed strong correlations across all four parameters, supporting that maximum-slice-based measurements broadly captured the parameter distribution observed in whole-tumor 3D analysis. However, the scatter points were not completely aligned with the identity line, indicating that the two approaches should not be considered interchangeable.

**
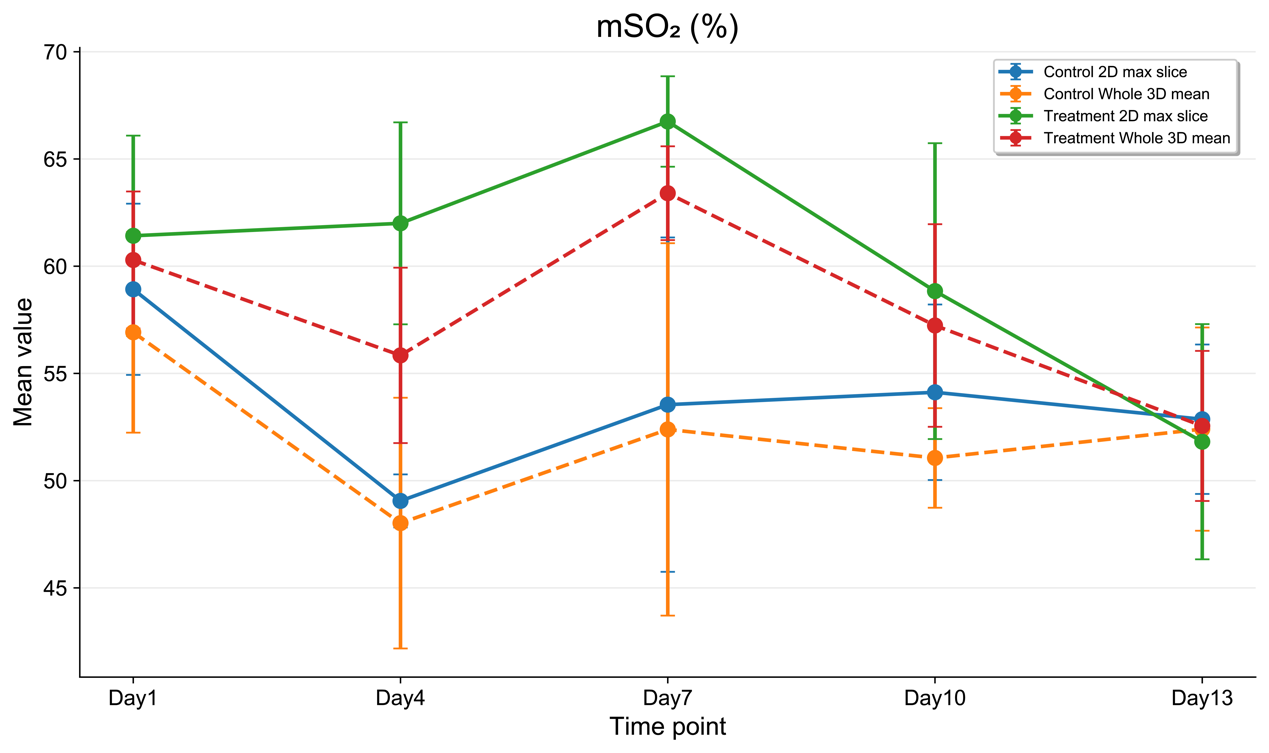
**

**Fig. S10. Descriptive longitudinal comparison of mSO₂ between 2D maximum-slice and whole-tumor 3D analyses.** Mean mSO₂ values from the 2D maximum cross-sectional slice and whole-tumor 3D measurements were plotted across Day 1, Day 4, Day 7, Day 10, and Day 13 for the control and CTX-treated groups. Data are shown as mean ± SD. Solid lines represent 2D maximum-slice measurements, whereas dashed lines represent whole-tumor 3D measurements. This descriptive comparison indicates that the main dynamic pattern of mSO₂, including the transient increase around Day 7 and the subsequent decline in the CTX-treated group, was broadly retained in the whole-tumor 3D analysis, while the two approaches were not identical in absolute values.

**
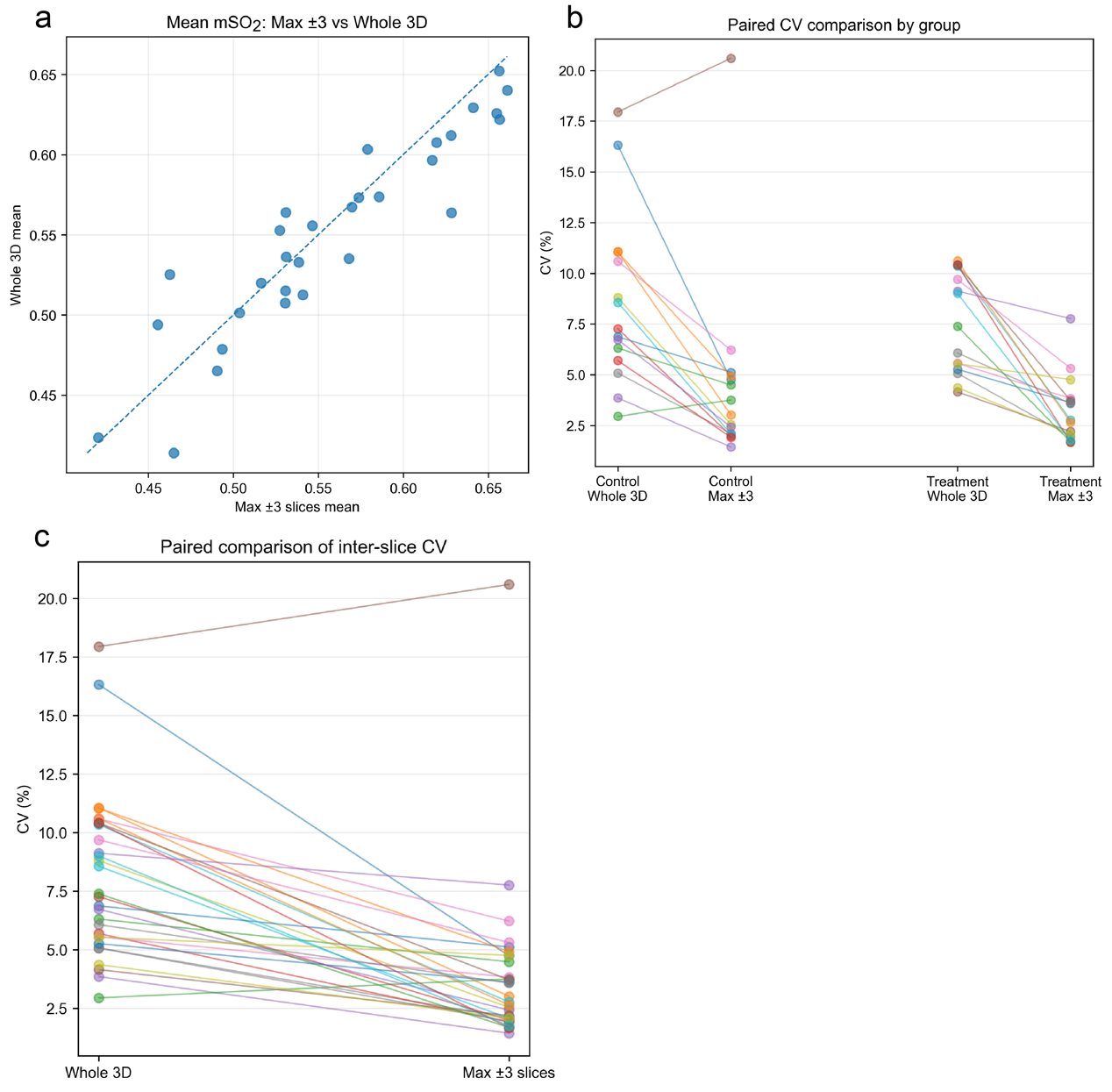
**

**Fig. S11. Inter-slice heterogeneity analysis of mSO₂ based on slice-by-slice data. (a) Paired scatter plot comparing the mean mSO₂ derived from the largest cross-sectional slice with adjacent ±3 slices and that derived from whole-tumor 3D slices. Each point represents one mouse-time point, and the dashed line indicates the identity line. (b) Paired comparison of inter-slice coefficient of variation (CV) between whole-tumor 3D slices and the largest slice ±3-slice range in the untreated control and CTX-treated groups. (c) Overall paired comparison of inter-slice CV across all analyzed mouse-time points. For each mouse-time point, CV was calculated from the raw slice-by-slice mSO₂ values either across all tumor-containing slices or across the largest slice with adjacent ±3 slices. Across 30 mouse-time points from representative mice, whole-tumor 3D slices showed a higher CV than the largest slice ±3-slice range (8.07 ± 3.46% vs. 3.89 ± 3.51%, P < 0.001), indicating greater inter-slice heterogeneity when the entire tumor volume was included.**

**Supplementary Table**

**Table S1. Comparison of inter-slice mSO₂ variability between whole-tumor 3D analysis and the maximum-slice ±3-slice approach**

| **Scope** | **Mice / mouse-time points** | **Whole-tumor 3D CV (%)** | **Maximum slice ±3 slices CV (%)** | **Mean difference, 3D − Max ±3 (%)** | ***P* value** |
| --- | --- | --- | --- | --- | --- |
| Overall | 6 mice / 30 mouse-time points | 8.07 ± 3.46 | 3.89 ± 3.51 | 4.18 | ＜0.001 |
| Control | 3 mice / 15 mouse-time points | 8.61 ± 4.24 | 4.49 ± 4.69 | 4.12 | ＜0.001 |
| Treatment | 3 mice / 15 mouse-time points | 7.54 ± 2.48 | 3.29 ± 1.68 | 4.24 | ＜0.001 |
